# Male mate choice for large gravid spots in a livebearing fish

**DOI:** 10.1093/beheco/arz156

**Published:** 2019-09-27

**Authors:** Hannah J P Ogden, Raïssa A de Boer, Alessandro Devigili, Charel Reuland, Ariel F Kahrl, John L Fitzpatrick

**Affiliations:** Department of Zoology: Ethology, Stockholm University, Svante Arrhenius väg, Stockholm, Sweden

**Keywords:** male choosiness, receptivity signals, sexual selection, sexual conflict

## Abstract

Male mate choice occurs in a wide range of species, and males can increase their reproductive success by distinguishing between females based on their fecundity (e.g., large body size) or their expected sperm competition risk (e.g., virgins). However, patterns of male mate choice could be mitigated by variation in female physiological receptivity, as males can benefit by directing their mating efforts toward females that are at a point in their reproductive cycle when fertilization probability is highest. Here, we perform three experiments to assess whether male mate choice is influenced by cues of female physiological receptivity, fecundity, or sperm competition risk in the pygmy halfbeak (*Dermogenys collettei*), a small livebearing fish. Female halfbeaks possess a “gravid spot”—an orange abdominal marking that is caused by pigmentation of the females’ skin and variation in embryo development and pigmentation during pregnancy. We show that gravid spot size increases toward parturition and is largest right before giving birth, independent of abdominal width or body size. Males consistently chose females with large gravid spots over females with small gravid spots. In contrast, males did not prefer larger females over smaller females or virgin females over mated females. As female halfbeaks store sperm prior to fertilizations, we suggest that males use the size of the gravid spot as a cue to direct their mating efforts to those females where the chance of fertilization is highest.

## Teaser

When is it a good time to mate? In fish that give birth to live young, females have a mark on their abdomen, a “gravid spot,” that changes in size over their reproductive cycle. Using a livebearing fish, the pygmy halfbeak, we show males pay attention to gravid spot size, going out of their way to court females with the largest gravid spots. We suggest that gravid spots provide information about female receptivity.

## INTRODUCTION

Selecting the right mate is a decision that can have a major impact on an animal’s fitness. Traditionally, females are considered the sex that should be choosy when it comes to mating because of their greater investment in gametes and offspring compared with males (Bateman 1948; Trivers 1972). However, males too can face substantial costs associated with mating in the form of energetically demanding courtship displays and investment in costly ejaculates (Partridge and Farquhar 1981; Dewsbury 1982; Nakatsuru and Kramer 1982; Cordts and Partridge 1996; Olsson et al. 1997). Males are, therefore, expected to exert mate choice whenever females vary in quality, the number of available mates exceeds the males’ mating capacity, and the benefits of being choosy outweigh the costs (Edward and Chapman 2011). Indeed, a growing number of studies across a range of taxa demonstrate that male mate choice is common (Amundsen 2000; Bonduriansky 2001; Schlupp 2018). Yet, relatively little is known about which female traits should be targeted by male mate choice (Schlupp 2018).

Male preferences during mate choice are typically studied in relation to female fecundity or mating status. More specifically, the female body size is often hypothesized to be an important trait in male mate choice due to the general link between body size and fecundity (Olsson 1993; Kraak and Bakker 1998; Byrne and Rice 2006; Liu et al. 2017). Males may also increase their reproductive success though preference for females where the risk and/or intensity of sperm competition is reduced, choosing either virgin or young females over mated or older females (Polak et al. 1998; Bonduriansky 2001). However, the expression of male preference for body size or mating status may be moderated by variation in female receptivity (Dixson 1983; Rowland et al. 1991; McLennan 1995; LeBas and Marshall 2000; Amundsen and Forsgren 2001). In many species, females exhibit cyclic changes in reproductive state (and behavioral and physiological receptivity) in accordance with their proximity to the next time of ovulation (Amundsen and Forsgren 2001; Baird 2004; Roberts et al. 2004). Males can, therefore, benefit by being attentive to cues of female reproductive states that allow them to bias mating efforts toward more receptive females (Kelso and Verrell 2002), irrespective of cues of female fecundity and/or mating status. However, few studies have focused on the importance of cues of receptivity in male mate choice and how they may be intertwined with cues of female fecundity and mating status.

Here, we investigate male mate choice in the pygmy halfbeak (*Dermogenys collettei*), a small tropical freshwater livebearing fish (Meisner 2001; Greven 2010). Although male investment into offspring is limited to the transfer of gametes, males invest substantially in testicular tissue (testes mass accounts for up to 6% of male body mass; unpublished data), suggesting that male mate choice may evolve in pygmy halfbeaks. Males are faced with a choice of courting numerous females present in mixed-sex groups that may vary in body size (a cue of fecundity), sperm competition risk, and receptivity. Female halfbeaks store sperm and give birth to broods in monthly cycles (Greven 2010). Males may, therefore, benefit by directing their courtship behaviors to females at points in their brood cycle when they are more receptive through increased mating success, preferential use of stored sperm, and/or increased fertilization success (Schlupp 2018). In particular, female halfbeaks display an orange abdominal marking called a “gravid spot.” Gravid spots vary in size among females (Ogden and Fitzpatrick 2019) and their appearance (as it is found in other livebearing fish) is the result of variation in pigmentation of the eggs and/or ovarian sac during pregnancy and of pigmentation of the females’ skin (Norazmi-Lokman et al. 2016). Gravid spots are hypothesized to provide information about the stage of embryo development and female fecundity (Norazmi-Lokman et al. 2016) and may present males with a clear cue of a female’s physiological receptivity (Ogden and Fitzpatrick 2019). As such, the gravid spot is hypothesized to be an important trait in male mate choice (Schlupp 2018). Yet, empirical tests of this hypothesis are scarce (but see Deaton 2008).

In this study, we characterize how the gravid spot changes over the female reproductive cycle in pygmy halfbeaks to determine if gravid spot size offers information that males can use when exerting mate choice. We then test whether the size of gravid spots, body size, and mating status (virgin or mated) of females influence patterns of male mating preference. We predict that changes in the size of the gravid spot relate to the brood cycle, that the gravid spot predicts female fecundity, and that males’ preference for females depends on the size of the gravid spot. Further, we predict that males should prefer large (over small) and virgin (over mated) females.

## METHODS

### Study species

**Figure 1:**
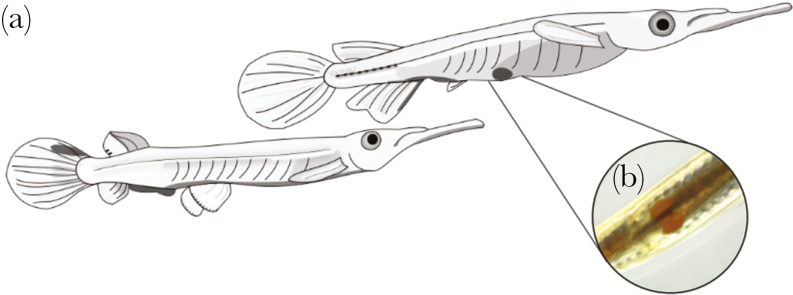
Courtship behavior and gravid spot size in pygmy halfbeaks. (a) A drawing of a male pygmy halfbeak “swimming under” a female, a behavior that occurs for an extended amount of time during courtship. During this swimming under behavior, males can visually access the “gravid spot,” here drawn as a gray spot in the pelvic region of the female. (b) A picture of a females’ ventral side shows the gravid spot as a marked orange coloration. Drawing by R.A.dB., picture by H.J.P.O.

Pygmy halfbeaks (*D. collettei*) are a small (<~4 cm) tropical fish found commonly in freshwater streams, rivers, and ponds in southern Peninsular Malaysia and Singapore (Meisner 2001; Nurul Farhana et al. 2018). Halfbeaks are an internally fertilizing, viviparous species that are characterized by their elongated lower jaws called a beak (giving this group of fish their name, i.e., “halfbeaks”). Halfbeaks are sexually dimorphic, both in terms of body size (females are larger) and coloration (males have more colorful fins). Like other species in the genus *Dermogenys*, pygmy halfbeaks are specialized surface feeders, with diets consisting predominantly of small insects (Meisner and Collette 1998). For example, in a closely related halfbeak species from central Thailand (*Dermogenys pusillus*), the majority of the halfbeaks diet consists of hymenopterans and dipterans (Ward-Campbell et al. 2005). Halfbeaks form large mixed-sex groups near the surface of the water where there are frequent courtship and agonistic interactions among and between males and females (Greven 2010). Within these mixed-sex groups, males spend a substantial amount of their time on courtship behaviors (Greven 2010; see Results). In particular, the courtship behavior of halfbeaks typically starts with males performing a “circling” behavior, in which the male approaches the female and swims around her head in a semicircular path (Greven 2010). Courtship continues when males perform a “swimming under” behavior, where the male swims under the female and positions himself ventrally posteriorly to the female so that his head is directly underneath the female’s genital pore (by no more than one body height; Figure 1a), allowing visual access to the gravid spot (Figure 1b). Swimming under is an important courtship behavior because mating can only occur when the male positions himself directly underneath the female, while the female remains stationary above the male. Moreover, swimming under represents a conspicuous, time-consuming component of male courtship behaviors in halfbeaks (Greven 2010; see Results), during which males must forgo feeding as they are not able to access the water surface when swimming under the female. Males may also use their beaks to express other courtship behaviors, including “nipping,” where males rapidly open and close their beak while directing this behavior toward the genital pore of the female, and “checking,” where males make physical contact with the female’s anterior region using their beak (Greven 2010). Females do not show obvious behavioral cues of receptivity during mating, rather females respond to male courtship by moving slowly or remaining motionless in the water column (Greven 2010). Following these oftentimes prolonged courtship bouts, males copulate with females by rapidly (~40–80 ms) flexing their body and making contact between their modified anal fin (andropodium) used for sperm transfer and female genital pore (Greven 2010). Females produce broods on a roughly monthly cycle and can store sperm for up to six breeding cycles following a single mating (C.R., personal observation).

### Study population and housing conditions

Focal fish were generated from adult halfbeaks obtained from a commercial supplier (Ruinemans Aquarium B.V., Montfoort, the Netherlands) and were kept in mixed-sex stock aquaria (ranging from 74 to 400 L) in groups of 20–50 individuals. To generate focal fish, gravid females (i.e., females with distended abdomens) were selected from the stock aquaria and isolated in 7.5-L tanks and monitored daily until they gave birth. Following birth, females were removed from the tank to prevent maternal infanticide. Offspring (i.e., fry) were reared in family groups, with a maximum of seven fry per tank. After the developing (thickening) andropodium on males could be identified, fry were kept together in single-sex groups of 20–30 individuals in 72–175-L tanks. Halfbeaks become sexually mature at ~4 months of age, and because males and females were separated before the fish reached sexual maturity, all fish were assumed to be virgins at the time of the experiments. All tanks were oxygenated and contained ~2 cm of gravel and plastic plants. Fish were fed twice daily with flake food and freeze-dried *Artemia* and once per week with previously frozen *Drosophila melanogaster*. The laboratory was maintained at 27 °C and had a 12:12 light:dark photoperiod.

### Quantifying variation in the gravid spot size over the female reproductive cycle

Females (*n* = 13) from mixed-sex stock tanks were isolated and monitored in 7.5-L tanks. The ventral side of females was photographed twice per week to measure female body length and gravid spot size. Females were placed into a clear plastic photography container (75 × 50 × 25 mm) filled with water from their own tank, and the photography chamber was placed on a plexiglass sheet held in place above a digital SLR camera (Canon EOS 600D, equipped with an EFS 18–135-mm lens) that was used to capture images. Females were typically in the photography chamber for less than 1 min before being returned to their tank. From these photos, standard body length (mm; distance between anterior point of the eye and caudal peduncle), gravid spot area (mm^2^), and abdomen width (mm) were measured using ImageJ (version 1.52a; Schneider et al. 2012). Female tanks were checked daily for the presence of fry. When fry were found, an additional photograph was taken the day after birth to assess whether parturition had any immediate effect on the gravid spot size. Because the gestation period of female halfbeaks in our laboratory is approximately 32 days (mean ± standard error (SE), 31.8 ± 0.6 days, *n* = 62 interbrood intervals), we continued to take photographs of females for 16 days after they had given birth, allowing temporal changes of the abdominal spot to be recorded over a full reproductive cycle.

### Experimental procedure

To evaluate male mate choice, males were presented with the simultaneous choice of two females in a free-swimming assay (experimental tank dimensions: 40 × 24 × 30 cm). All tanks were filled to a depth of 12 cm, contained three pieces of plastic plant and ~1-cm layer of gravel, and were oxygenated. In all trials, a transparent plexiglass cylinder (20-cm diameter) was placed in the center of the experimental tank and two females were then added to the tank outside the plexiglass cylinder. Trials were performed blind by distinguishing females based on phenotypic differences (e.g., beak morphology) and arbitrarily referring to them as “Female 1” or “Female 2” during the trial. A focal male was then placed inside the transparent plexiglass cylinders, allowing the male to see, but not physically interact with, the females. Experimental tanks were then left undisturbed during a 1.5-h habituation period. After the habituation period, the plexiglass cylinder was lifted using a pulley system, allowing males and females to interact. To ensure males made an informed decision, trials were only included in analyses if males interacted with both females (details on how this exclusion criteria influenced sample sizes are provided below). Observations started as soon as the male interacted with one of the females and lasted for 20 min.

Male mate choice was assessed in three separate experiments. In Experiment 1, males were presented with two size-matched females that differed in gravid spot area (large vs. small gravid spots). In Experiment 2, males were given the choice of females that differed in body size (large vs. small females). Because the aim of Experiment 2 was to assess male responses to variation in female body size, we did not attempt to control for natural variation in spot size among females in this experiment. Moreover, experimentally manipulating the size of the gravid spot is challenging as we have not yet established a method to modify the size of the spot without influencing either the female or the developing embryos. Finally, in Experiment 3, males were presented with two size-matched females that differed in mating status (virgin vs. mated females) but not body length and spot size. Male mate choice behavior was recorded during the trials. Male mate choice behavior was quantified as the duration of time (seconds) males spent swimming under females, the sum of all male courtship behaviors performed during the observation period (circling, nipping, checking, and the number of times a male swam under a female, henceforth called “total courtship count”), and the number of copulations (note that we assumed males were successful at transferring sperm during copulations). After the mate choice experiments, fish were photographed under standard conditions. All fish were digitally photographed under a Leica S9i stereo microscope using LAS X software (Leica, Germany). Before taking photographs, each fish was sedated in a benzocaine solution (600 μl stock solution per 1l H_2_O, where stock solution = 150 μl benzocaine per 1 mL ethanol). In both sexes we measured standard body length (mm) by photographing the fish on their left-lateral side, with a scale included in every image. For females, an additional photograph of the ventral surface was taken to measure the gravid spot area (mm^2^; Figure 1B). All photographs were analyzed in ImageJ (version 1.52a; Schneider et al. 2012).

### Experiment 1: large versus small gravid spots

We tested if male preference was related to the size of female’s gravid spot using the 13 females whose temporal variations in gravid spot size had been measured over a full reproductive cycle (see Quantifying variation in the gravid spot size over the female reproductive cycle). Because gravid spots size changes over a brood cycle (see Results), spot size was measured no more than 1 day before the trial was conducted with that female. We then used the 13 females to generate 10 unique pairings between females that were of similar body size but with interindividual variation in spot size area. By design, gravid spot area was significantly larger in the females assigned as the large gravid spot stimuli (mean ± SE, 3.06 ± 0.17 mm^2^) than in females assigned as the small gravid spot stimuli (mean ± SE, 1.10 ± 0.11 mm^2^, *t* = 8.20, *P* < 0.0001), but female body length did not differ between large gravid spot (mean ± SE, 29.36 ± 0.20 mm) and small gravid spot (mean ± SE, 27.81 ± 0.30 mm) females (*t* = 1.65, *P* = 0.10). Due to restricted availability of females with known brood dates, each unique pairing was reused once (with the exception of one pairing where the gravid spots between females became consistently too similar in size for them to be used again). A total of 23 males were used (mean ± SE [range] male body length: 22.69 ± 0.09 mm [21.74–23.91 mm]; mean ± SE [range] difference between male and stimuli female body length: 5.9 ± 0.26 mm [2.63–8.58 mm]). Four replicates were excluded because the male only interacted with one female (only the small gravid spot female in *n* = 3 trials and only the large gravid spot female in *n* = 1 trial), bringing the final sample size used in analyses to 19 replicates.

### Experiment 2: large versus small body size

We tested if male preference was influenced by female body size by presenting males with two virgin females that were selected based on a visual distinction of a difference in body size. Female body length was measured after the trials to quantify the difference in female size generated from the initial visual classification. There was a difference in body length of (mean ± SE) 5.02 ± 0.52 mm between large (mean ± SE, 29.39 ± 0.65 mm) and small (mean ± SE, 24.37 ± 0.35 mm) females (*t* = 6.62, *P* < 0.0001). Spot size was positively related to body length in the females used in this experiment (linear model: *t* = 3.67, *P* < 0.001), making it challenging to disentangle the effects of body size and spot size in halfbeaks. Indeed, the large females (mean ± SE, 2.26 ± 0.16 mm^2^) had a larger gravid spot area than small females (mean ± SE, 1.39 ± 0.15 mm^2^; *t* = 4.17, *P* = 0.0001). Nevertheless, body length explained only 21% of variation in spot size among females and, in 4 of the 24 replicates, the spot size was larger in the smaller female used in the replicate. It was tested whether this difference in spot size affected male mate choice behavior (see Statistical analyses). Trials were conducted on 33 males (mean ± SE [range] male body length: 23.72 ± 0.20 mm [21.66–24.74 mm]; mean ± SE [range] difference between male and stimuli female body length: 3.27 ± 0.52 mm [−2.44–14.27 mm]). Nine replicates were excluded because the male interacted with none (*n* = 3) or only one of the females (*n* = 6; in three of the trials, the male only interacted with the small female and, in the other three, only with the large female), bringing the final sample size used in the analysis to 24 replicates.

### Experiment 3: virgin versus mated females

We assessed whether male mate choice was influenced by female reproductive status (i.e., whether the female was virgin or mated). In these trials, a focal male was presented with one virgin and one mated female that were visually size matched on the basis of body length (mean ± SE difference: 0.56 ± 0.6 mm). Body length did not differ between virgin (mean ± SE, 30.43 ± 1.15 mm) and mated (mean ± SE, 30.98 ± 1.12 mm) females (*t* = 0.31, *P* = 0.76). There was no difference in gravid spot area between virgin (mean ± SE, 1.96 ± 0.23 mm^2^) and mated (mean ± SE, 2.23 ± 0.25 mm^2^) females (*t* = −0.58, *P* = 0.56). Mated females were not used if they had given birth less than a week before the experiment. Five mated females were used within two different replicates to facilitate size matching with virgin females. However, as these mated females were paired with a different virgin female when they were reused, all replicates were treated as independent in subsequent analyses. Twenty-four males were assessed (mean ± SE [range] male body length: 23.58 ± 0.29 mm [19.78–26.82 mm]; mean ± SE [range] difference between male and stimuli female body length: 6.8 ± 0.95 mm [−0.59–21.12 mm]). Nine replicates were excluded because the male interacted with none (*n* = 2) or one of the females (*n* = 7; in five of the trials, the male only interacted with the virgin female and, in the other two, only with the mated female). The final sample size in our analysis was 15 replicates.

### Statistical analyses

All analyses were completed using R version 3.4.4 (R Core Development Team 2019) using the *lm* function or functions in the *lme4* package (Bates et al. 2015). Statistical significance of the models described below was assessed using the Anova function in the *car* package.

### Quantifying variation in the gravid spot size over the female reproductive cycle

To determine gravid spot size changes over a brood cycle, data on gravid spot area was split into the 16 days before giving birth and the 16 days after giving birth to meet the requirements for the use of linear models. Two separate linear mixed models (LMMs; one for changes in gravid spot size before birth, one for after birth) were then used to test whether gravid spot size was dependent on time in the brood cycle. In both models, the log-transformed gravid spot size was included as the response variable, time relative to giving birth as a fixed effect, and body length was included as a covariate. Female identity was included as a random effect to account for the repeated measures within females. In addition, gravid spot size the day after giving birth was compared with the measurement of gravid spot size taken closest to giving birth for each female (−3 days, *n* = 6; −2 days, *n* = 4; −1 day, *n* = 3 females). An LMM was constructed with the natural log-transformed gravid spot size as the response variable, time as a fixed effect, body length as a covariate, and fish identity as a random effect. To test if changes in gravid spot size were independent of changes in abdominal width (due to pregnancy), the analyses were redone with abdominal width instead of body length included as covariate. This modeling approach was used to avoid statistical issues that arise from the inclusion of ratios (e.g., spot size/body length) in models (Tomkins and Simmons 2002) or the use of residual values from linear regressions, which produces biased parameter estimates when correlation exists between predictor variables (Freckleton 2001). To test if gravid spot size on the day closest to giving birth predicted brood size, we used an LMM with brood size as the response variable and the gravid spot size and body length as the explanatory variables.

### Male mate choice experiments

To test whether male mate choice was influenced by female body length, gravid spot size, or mating status, LMMs, and generalized LMMs (GLMMs) were used. The three parameters of male mate choice (duration of swimming under, total courtship count, and number of copulations) were included as response variables in separate models for each experiment. Duration of swimming under the female was log-transformed (to achieve a normal distribution) and assessed using LMMs, whereas count data (i.e., courtship behaviors and copulations) were assessed using GLMMs fitted with a Poisson error distribution. The different experiments were analyzed separately and, in each LMM or GLMM, female experimental treatment (small/large body size, small/large gravid spot, and virgin/mated) was entered as a fixed effect. When assessing the duration of time under a female in LMMs, trial number (i.e., male identity) was added as a random effect because courtship behaviors toward each female within a trial are not independent measures. For LMMs in Experiment 1 (large vs. small gravid spots), pairs of females were used twice and, therefore, “pair identity” was added as a random effect in these models. For LMMs in Experiment 2 (large vs. small body size), the random effect variance in trial number (i.e., male identity) was estimated to be nearly zero (i.e., noninformative) and was, therefore, dropped from the model. Therefore, duration of time under a female was assessed using a linear model for Experiment 2. In GLMMs, observation number (a unique ID for all females among replicates) was added to all models corrected for overdispersion in the data. Once again, in GLMM models, the random effect variance in trial number was low and, therefore, GLMMs did not include trial number as a random effect. In each of the three experiments, we also assessed if the difference in body length between females and males influenced male courtship and mating behaviors by adding these variables as covariates to the models described above.

In the body size experiment (Experiment 2), we did not control for natural variation in spot size (see above). Therefore, we assessed if the difference in gravid spot size between large and small females had an effect on male mate choice behavior. Stimuli females within a trial were randomly assigned “Female 1” and “Female 2” to disentangle the difference in body length from the difference in gravid spot area. The difference in gravid spot area (gravid spot area Female 1—gravid spot area Female 2) was then included as covariate in a model. Strength of preference (SOP) scores were used to estimate relative male preference for either female within each trial. SOP scores were calculated as courtship behaviors directed toward Female 1/total courtship behaviors directed toward either female. Because SOP scores could not be calculated for replicates where the specified behavior did not occur, the number of replicates varied among models. SOP scores were analyzed as response variables in generalized linear models (GLMs; separate models for swimming under duration, courtship count, and copulation count) with a quasibinomial error distribution (as SOP ranged between 0 and 1).

Effect sizes (Hedges’ g) were calculated in order to quantify the magnitude of the differences (Nakagawa and Cuthill 2007; Cumming 2011) in male courtship behavior depending on variation in female body size, spot size, and mating status. To account for the paired design, an unbiased estimate of the effect size (Hedges’ g) was determined using $g=\frac{M_{dif\;f}}{S_{av}}J$ in which $J=1-\frac{3}{4\left(N-1\right)-1}$ and $S_{av}=\sqrt{({S_{1}}^{2}+{S_{2}}^{2})/2}$ (Cumming 2011). The 95% confidence intervals (CIs) were determined with an iterative approach using the noncentral *t*-distribution with the aid of Exploratory Software for Confidence Intervals (Cumming and Calin-Jageman 2012).

### Variation in male mating behaviors

To assess and compare variation in male mate choice behaviors, we summed all mate choice behaviors observed during the behavioral trial (i.e., regardless of which female it was directed toward) to obtain a total duration or count of behaviors for each male. To compare mate choice behaviors among experiments we used linear models to assess the duration of time under a female and GLMs with Poisson error distributions to assess count data (note that, in these models, the male was the unit of replication and, therefore, tank and pairing of females were not included as random factors). The relationship between male body length and the expression of mate choice behaviors was assessed using linear models.

### Ethical note

To decrease the number of animals used, stimuli females were reused between size-based and mating status free-swimming assays. Experiments were approved by the Swedish Board of Agriculture (Jordbruksverket permit number 2393-2018).

## RESULTS

### Variation in gravid spot size over the reproductive cycle

**Figure 2:**
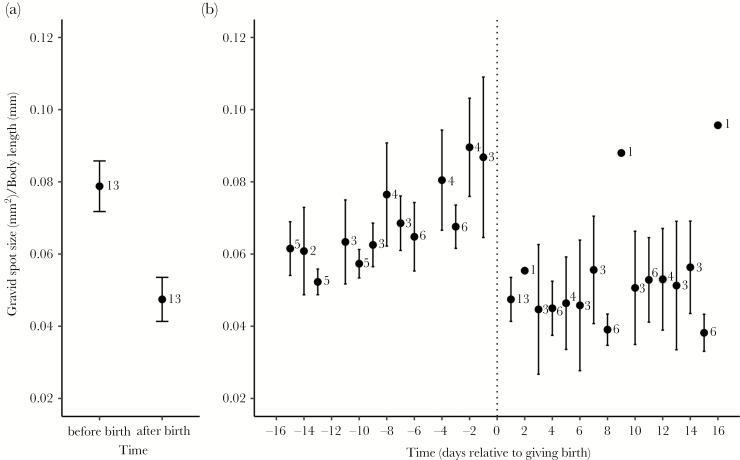
Variation in gravid spot size over the brood cycle. (a) Gravid spot size relative to body length is larger on the day closest to giving birth than on the day after parturition. (b) During a brood cycle, gravid spot size gradually increases toward the day of parturition and shows a steep drop in size after parturition. Error bars represent standard error of the mean. Numbers note the sample size for each data point. These data are for illustrative purposes only; all conclusions from the analyses are based on linear models where female body length was treated as a covariate.

Gravid spot area differed markedly before and after birth. Spot area in the day(s) immediately preceding birth (mean ± SE, 1.97 ± 0.17 mm^2^) was ~60% larger than spot area on the day after birth (mean ± SE, 1.21 ± 0.15 mm^2^; χ ^2^ = 12.23, *P* = 0.0005; Figure 2a). Gravid spot size increased in the 16 days leading up to giving birth (χ ^2^ = 34.06, *P* < 0.0001; Figure 2b). In contrast, gravid spot area showed little change during the 16 days after giving birth (χ ^2^ = 2.47, *P* = 0.06; Figure 2b). Larger gravid spots, therefore, indicate where females are in their reproductive cycle. When accounting for abdominal width (instead of body length), the results regarding the temporal changes in gravid spot size remained the same (before birth: χ ^2^ = 38.92, *P* < 0.0001; after birth: χ ^2^ = 0.87, *P* = 0.35). There was no relation between brood size and the size of the gravid spot on the day closest to giving birth (*F*_1,10_ = 0.10, *P* = 0.76) nor between brood size and body length (*F*_1,10_ = 2.96, *P* = 0.12).

### Male mate choice experiments

**Figure 3:**
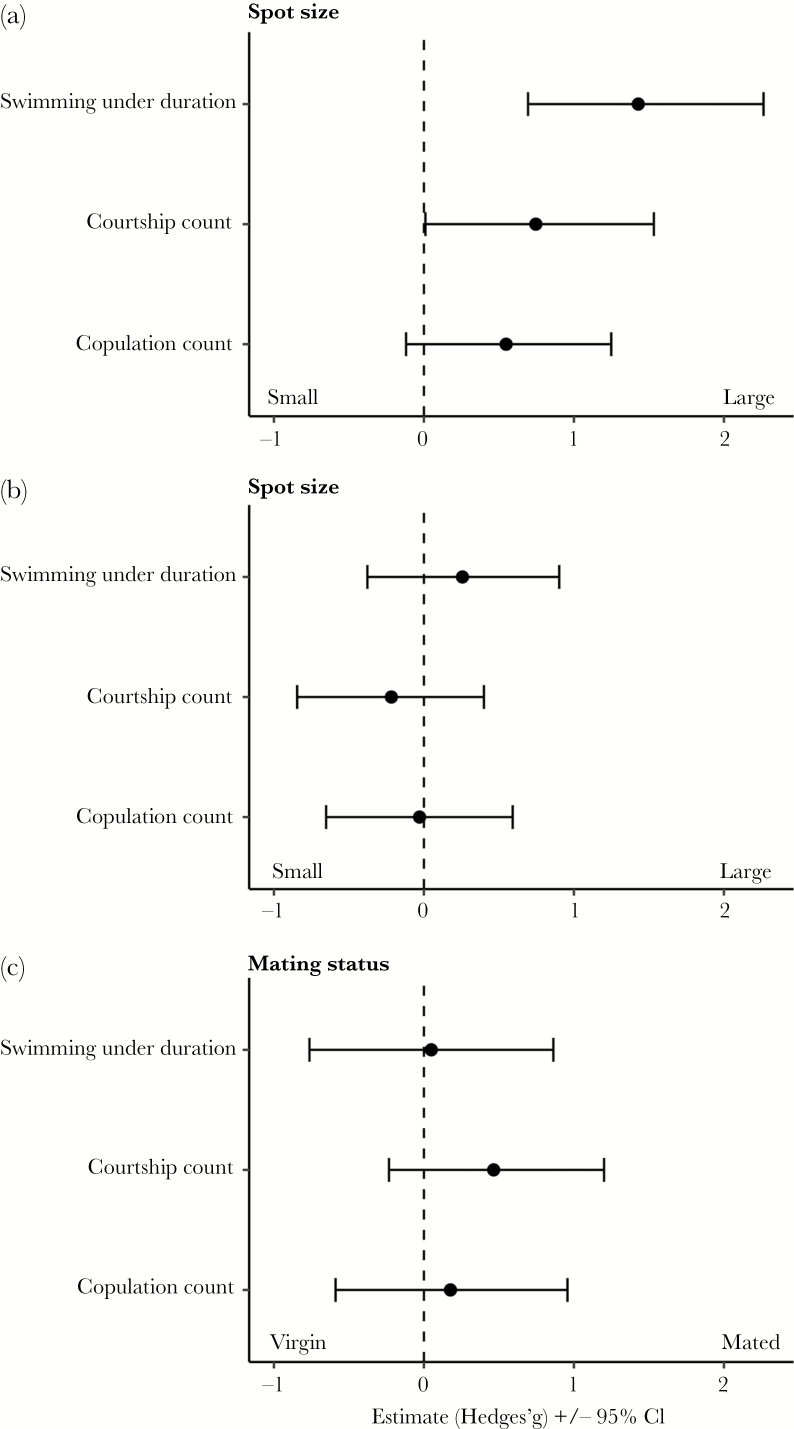
The effect size (±95% CI) of the difference in male courtship behaviors (swimming under duration, courtship count, and copulation count) directed toward females that varied in (a) gravid spot size, (b) body size, and (c) mating status. There was a consistent effect of gravid spot size on the occurrence of male courtship, with males preferring females that have large gravid spots. There were no notable differences in male mate choice when males were presented with females that differed in body size or mating status.

When presented simultaneously with two length-matched females that differed in the size of their gravid spot (Experiment 1), males directed more courtship behaviors toward females with larger gravid spots than females with smaller gravid spots. Males swam under females with larger gravid spots 5.5 times longer than under females with smaller gravid spots and directed nearly twice as much total courtship count behaviors toward females with larger gravid spots (Figure 3a; Table 1). The limited number of copulations observed during the trials (see Table 1) provided limited scope to detect differences in copulation number between treatments. Nevertheless, males copulated with females with larger spots twice as often as with females with smaller gravid spots, although this effect was marginal (Figure 3a; Table 1).

**Table 1:** The effect of stimuli females that differed in: 1) gravid spot size, 2) body size, and 3) mating status on male mate choice behaviors (swimming under, courtship, and copulation) in halfbeaks. The mean (±SE) behavior duration (swimming under) and count (courtship and copulation) that males directed at the stimuli females are presented for each experiment

	Behavior	Mean behaviors (±SE)		Predictor	*n*	χ ^2^	*P*
1)	Experiment 1: gravid spots	Large spot	Small spot				
	Swimming under duration	265.80 ± 45.41	48.74 ± 12.85	Spot size	19	**22.91**	**<0.001**
	Total courtship count	27.79 ± 4.87	14.79 ± 2.36	Spot size	19	**5.85**	**0.02**
	Copulation count	2 ± 0.52	1 ± 0.23	Spot size	19	2.99	0.08
2)	Experiment 2: body size	Large body	Small body				
	Swimming under duration*	208.38 ± 51.74	142 ± 49.89	Body size	24	**5.48**	**0.02**
	Total courtship count	14.67 ± 2.30	17.50 ± 2.81	Body size	24	0.35	0.55
	Copulation count	0.92 ± 0.28	0.96 ± 0.29	Body size	24	0.01	0.91
3)	Experiment 3: mating status	Virgin	Mated				
	Swimming under duration	249.33 ± 76.17	264.07 ± 68.18	Mating status	15	0.99	0.32
	Total courtship count	13.40 ± 3.04	22.67 ± 6.17	Mating status	15	1.35	0.25
	Copulation count	1 ± 0.32	1.40 ± 0.71	Mating status	15	0.07	0.80

The * indicates models where the random effect variance was too low to estimate and was removed from the model (note that test statistics, in this case, are *F*-values rather than χ ^2^ values). Significant results are indicated in bold.

When offered the simultaneous choice of a large and small female (Experiment 2), males spent more time swimming under larger females than smaller females (Table 1), although the effect size indicated that the magnitude of this difference was negligible (Figure 3b). Males did not direct more total courtship count behaviors to larger females, nor did they attempt to copulate with larger females more often than with smaller females (Figure 3b; Table 1). Gravid spot size varied with female body length in Experiment 2 (see Methods). Therefore, the difference in female body size introduced by the experimental treatment generated variance in gravid spot area among treatments. We tested if the difference in gravid spot size between large and small females influenced male mate choice behaviors. Consistent with the results from Experiment 1, males demonstrated a greater SOP in swimming under behavior as the magnitude of difference between females’ gravid spots increased, with males exerting stronger preference for females with larger gravid spots (χ ^2^ = 14.8, *P* = 0.0002). The SOP for total courtship count (χ ^2^ = 0.89, *P* = 0.30) and copulation count (χ ^2^ = 0.27, *P* = 0.60) was not affected by the magnitude of difference in gravid spot area between large and small females.

When males were presented with two size-matched females that differed in mating status (Experiment 3), the duration of time males spent swimming under females, the total courtship count, and the number of copulations did not differ between virgin and mated females (Figure 3C; Table 1).

To determine if variation in male mating preferences were influenced by the relative size between males and females, we performed an additional set of analyses where we assessed the experimental treatment effect from each of the three experiments separately (as above) but included the difference in body length between the stimuli females and focal male as a covariate in the models. In Experiment 1 (large vs. small gravid spots) and Experiment 2 (large vs. small body size), the size difference between female and male body length was not related to any of the male mate choice behaviors examined (Supplementary Table S1). In Experiment 3 (virgin vs. mated females), there was a negative relationship between size difference between females and males and total courtship count, such that males courted females more when the size difference between the sexes was low (Supplementary Table S1). Other mate choice behaviors (swimming under and number of copulations) were not related with the difference in length between females and males in Experiment 3 (Supplementary Table S1). In all of these additional models, the main treatment effects of gravid spot size, body length and mating status remain qualitatively consistent with the experimental treatment effects reported in Table 1, suggesting that these effects are not driven by confounding variance in the difference between female and male body sizes.

### Variation in male mating behaviors

The total (i.e., the sum of all behaviors directed at both stimuli females) duration and number of mate choice behaviors varied among males. Across all observations, males spent roughly one third of the time during behavioral observations swimming under either of the females (mean total swimming under duration ± SE: 31.74 ± 3.21%, range: 1.58–88.58%). The average total number of courtship behaviors displayed by males during the observation period was 36.59 (±2.74, range: 4–90) and the average total number of copulations in behavioral trials was 2.26 (±0.3, range: 0–11). However, the total duration of time males spent swimming under a female (*F*_*2,55*_ = 1.87, *P* = 0.16), the total courtship count (χ ^2^ = 2.67, *P* = 0.26), and the number of copulations (χ ^2^ = 2.67, *P* = 0.26) did not differ among the three experiments. Male body length was not related to the total duration or number of any of the behaviors we assessed across the three experiments (duration under female: linear model, *t* = −0.04, *P* = 0.97; total courtship count, χ ^2^ = 0.95, *P* = 0.33; number of copulations, χ ^2^ = 0.002, *P* = 0.96).

## DISCUSSION

In this study, we examined male mate choice for gravid spot size, body size, and mating status in halfbeak fish. We first showed that the size of the female gravid spot varied across the monthly brood cycle, reaching a maximum size immediately before females gave birth and dramatically reducing in size after parturition. We then showed that male halfbeaks directed more courtship behaviors to females with larger gravid spots when presented with females with different-sized gravid spots but similar body sizes (Experiment 1). Specifically, every stage of halfbeak mate choice behavior, from males swimming under females to performing courtship behaviors, leading to copulations (albeit a statistical trend), was exaggerated when males were presented with females that had larger gravid spots. Moreover, when presented with large and small females (Experiment 2), the males’ preference depended on variation in gravid spot size, whereas body size was irrelevant for male mate choice. In contrast, males did not show preference when females were different concerning their mating status but had no variation in spot size (Experiment 3).

Our findings indicate that male mate preference was specifically based on the size of the gravid spot in accordance with a recent suggestion (Schlupp 2018). The appearance of gravid spots in livebearing fish is not merely the result of pigmentation (e.g., carotenoids, Amundsen and Forsgren 2001) of the females’ skin but is also a physiological byproduct of pregnancy (Norazmi-Lokman et al. 2016). Therefore, gravid spots are not considered a trait that functions as a female ornament. Instead, the gravid spot offers cues of embryo development and fecundity (Norazmi-Lokman et al. 2016) and/or provides the male with information on the females’ brood cycle (this study). This bears resemblance to visual cues that advertise female reproductive state or receptivity that are found in other animals (e.g., primates (Dixson 1983), chameleons (Kelso and Verrell 2002), lizards (LeBas and Marshall 2000; Belliure et al. 2018), and oviparous fish (Rowland et al. 1991; McLennan 1995)). In halfbeaks, males can easily access this visual cue because they position themselves directly under the female during courtship (Figure 1). Male preference for females with large gravid spots likely comes at a cost. Across our experiments, males spent roughly one third (and as much as 88%) of their time courting females by positioning themselves under the female (i.e., swimming under). Males swam under females with larger gravid spots for more than five times as long as females with smaller gravid spots. Such male preference for larger gravid spots likely generates an energetic cost on males, as they expend energy on courtship and are unable to feed during these prolonged courtship bouts. By preferentially choosing females with large gravid spots, male halfbeaks select for females that are close to parturition. The adaptive significance of this remains to be resolved, and below we provide some suggestions.

The most straightforward interpretation of our results is that males prefer females with large gravid spots because these females are most physiologically receptive to fertilizations. As halfbeaks live in large mixed-sex groups, where males will regularly encounter females at various stages of their brood cycle, it would benefit males to direct their investment in courtship behaviors to those females where the probability of mating success is highest. Because females show no obvious behavioral cues of receptivity (Greven 2010), males may rely on physiological cues that provide information on where a female is in her reproductive cycle. Thus, we hypothesize that male preference for females with large gravid spots indicates that copulations immediately preceding parturition leads to preferential sperm use during fertilizations in halfbeaks. Such preferential sperm use could also influence a males’ postcopulatory success in sperm competition among rival males. Nonvirgin female halfbeaks store sperm (Greven 2010) and likely fertilize their broods after parturition. If halfbeaks show last male sperm precedence, as is often the case in other internal fertilizers (e.g., livebearing fish (Evans and Magurran 2001; but see Ala-Honkola et al. 2010; Magris et al. 2017), marsupials (Kraaijeveld-Smit et al. 2002), and insects and birds (Birkhead and Hunter 1990)), the male may benefit by using cues advertising when females will give birth to increase their competitive success during postparturition fertilizations. However, evaluating this potential requires a better understanding of sperm precedence and egg fertilization patterns in halfbeaks, which necessitates the development of genetic tools to address these questions in this species.

The aforementioned suggestions do not preclude the possibility that the gravid spot holds some cue to female quality. For example, in mosquitofish (*Gambusia holbrooki*) the size and intensity of the gravid spot correlated with brood size (Norazmi-Lokman et al. 2016). Visual cues that are simultaneously related to the reproductive cycle as well as female quality are found in other animals too, with males adjusting their courtship behavior toward females expressing conspicuous visual cues that indicate quality in these species (e.g., baboons (Domb and Pagel 2001) and cichlid fish (Baldauf et al. 2011)). However, we did not find a correlation between the size of the gravid spot and brood size in our study. Nevertheless, it is worth exploring further if spot size is related to other aspects of female fecundity or quality in halfbeaks (e.g., survival of the offspring and number of broods).

Contrary to theoretical expectations and the general pattern observed in a wide range of taxa (Olsson 1993; Kraak and Bakker 1998; Polak et al. 1998; Bonduriansky 2001; Byrne and Rice 2006; Liu et al. 2017), male halfbeaks did not preferentially direct mate choice behaviors toward either females with larger body size or to virgin females. Male preference for large body size is commonly explained by the fitness benefits gained from larger females being able to carry more eggs and, thus, producing more offspring (Bonduriansky 2001; Edward and Chapman 2011). However, many traits that may act as cues are associated with body size, and the abundance of studies demonstrating size-dependent male mate choice may be because preference for larger females is the most straightforward hypothesis to test (Schlupp 2018). Furthermore, the relationship between fecundity and body size is by no means uniform (Schlupp 2018). In the females sampled in this study, body size did not predict brood size. Instead, our results suggest that males only care about body size inasmuch as it predicts gravid spot size. Likewise, the lack of preference for virgin females could arise from a number of alternative explanations. First, males may need to observe competing males around the females and/or see them mating to pick up on cues of female mating status (Dosen and Montgomerie 2004). Second, if there is a high probability that halfbeak females will mate with multiple males, then virgin females may not represent an attractive mating opportunity, particularly if there is last male sperm precedence. Our findings, therefore, highlight the importance of considering multiple potential cues simultaneously, which may provide relevant information to males when exercising mate choice.

Altogether, the findings of this study suggest that large gravid spots are perhaps the most important feature relied on during mate choice in this species. Our study finds that body size was a less important trait than expected and that preference for large females only occurred because they had larger gravid spots. We also found no evidence that virgin females are preferred in this species, but this may be because males need other cues to get this information. In addition, given that halfbeaks live in large groups and females store sperm, males may benefit by focusing on signals that indicate receptivity since the chances of encountering a virgin are low. In addition, from the females’ point of view, there may be benefits of signaling their reproductive state. Male halfbeaks harass females relentlessly (Greven 2010), and it would benefit females if they can limit (potentially costly) male harassment to specific periods of the brood cycle (e.g., Amundsen and Forsgren 2001; Belliure et al. 2018). Assessing how cues of female receptivity interact with the potential for sexual conflict stands out as an exciting avenue to explore.

## FUNDING

This work was supported by the Knut and Alice Wallenberg Academy Fellowship to J.L.F., Carl Tryggers Stiftelse (17:152 to R.A.dB.), Wenner-Gren (to A.D.), and the Swedish Research Council (2017–04680).

## Supplementary Material

arz156_suppl_Supplementary_Material

## Notes

We thank two anonymous reviewers for their extremely useful comments on an earlier version of the manuscript.

Data accessibility: Analyses reported in this article can be reproduced using the data provided by Ogden et al. (2019).
