# Supplementary material for: Male mate choice for large gravid spots in a livebearing fish
Source: Behav Ecol. 2019 Sep 27;31(1):63–72. doi: 10.1093/beheco/arz156 (PMC7191251; doi:10.1093/beheco/arz156)
Supplement: arz156_suppl_Supplementary_Material [file arz156_suppl_supplementary_material.docx]

**Supplementary Material**

**Male mate choice for large gravid spots in a livebearing fish**

**Table S1:** The effect of stimuli females that differed in: A) gravid spot size, B) body size and C) mating status on male mate choice behaviours (swimming under, courtship, copulation) in halfbeaks in models that accounted for the difference in length between females and males. To account for variation in female and male body length among replicates, we included the difference in body length between the stimuli females and focal males in each model. The * indicates models where the random effect variance was too low to estimate and was removed from the model (note that test statistics in this case are *F*-values rather than χ^2^ values). Significant results are indicated in bold.

|  | Behaviour | Predictor | n | χ^2^ | *P* |
| --- | --- | --- | --- | --- | --- |
| A) | ***Experiment 1: Gravid spots*** |  |  |  |  |
|  | Swimming under duration | Spot size (large vs. small) | 19 | **14.42** | **<0.001** |
|  |  | Female-male size difference |  | 0.04 | 0.83 |
|  | Total courtship count | Spot size (large vs. small) | 19 | **8.33** | **0.004** |
|  |  | Female-male size difference |  | 2.14 | 0.14 |
|  | Copulation count | Spot size (large vs. small) | 19 | 2.56 | 0.11 |
|  |  | Female-male size difference |  | 0.03 | 0.86 |
| B) | ***Experiment 2: Body size*** |  |  |  |  |
|  | Swimming under duration* | Body size (large vs. small) | 24 | 2.31 | 0.13 |
|  |  | Female-male size difference |  | 0.01 | 0.91 |
|  | Total courtship count | Body size (large vs. small) | 24 | 0.19 | 0.66 |
|  |  | Female-male size difference |  | 1.51 | 0.22 |
|  | Copulation count | Body size (large vs. small) | 24 | 0.06 | 0.81 |
|  |  | Female-male size difference |  | 0.19 | 0.66 |
| C) | ***Experiment 3: Mating status*** |  |  |  |  |
|  | Swimming under duration | Mating status (virgin vs. mated) | 15 | 1.48 | 0.22 |
|  |  | Female-male size difference |  | 0.02 | 0.88 |
|  | Total courtship count | Mating status (virgin vs. mated) | 15 | 2.51 | 0.11 |
|  |  | Female-male size difference |  | **6.49** | **0.01** |
|  | Copulation count | Mating status (virgin vs. mated) | 15 | 0.40 | 0.53 |
|  |  | Female-male size difference |  | 1.95 | 0.16 |
